# A Journey through the Minefield of the Discovery and Characterization of Latency-Related RNA/Latency-Associated Transcript

**DOI:** 10.3390/v16101562

**Published:** 2024-09-30

**Authors:** Homayon Ghiasi

**Affiliations:** Center for Neurobiology & Vaccine Development, Ophthalmology Research, Department of Surgery, Cedars-Sinai Burns & Allen Research Institute, CSMC—SSB3, 8700 Beverly Blvd, Los Angeles, CA 90048, USA; ghiasih@cshs.org

**Keywords:** HSV-1, latency reactivation cycle, latency-related RNA (LR-RNA), latency-associated transcript (LAT), apoptosis, CD8^+^ T cells, IFNγ, exhaustion, single-cell analyses

## Abstract

Scientific knowledge evolves in small steps, with occasional backsteps to correct inaccuracies, all occurring within a competitive environment. This perspective for the first time looks at the history of latency-related RNA (LR-RNA) that was later renamed latency-associated transcript (LAT). At the 1986 International Herpesvirus Workshop (IHW) meeting in Leeds, England, Daniel L Rock and Anthony B Nesburn first reported the discovery of human herpes virus 1 (HSV-1) latency-related (LR) RNA that is antisense to ICP0. Less than a month after the IHW meeting, a paper was submitted to *Science* magazine and 8 months later appeared in print thanking “D. Rock for suggesting RNA complementary to the ICP0 message may be present in latently infected cells”. This perspective is not a review of the LAT literature but intends to clarify the timeline of LAT discovery and subsequent breakthroughs such as reactivation, apoptosis, CD8^+^ T cell exhaustion, and LAT expression in different cell types detected during latency. While many review articles have been written about LAT since 1987, the most comprehensive and balanced review about LAT was written by Dr. David Bloom’s group. In this overview, I will discuss our original collaboration with Dr. Dan Rock and subsequent work that our group performed, which is still ongoing. Finally, I will discuss the controversies associated with LAT from its inception to current times.

## 1. Introduction

Herpes simplex virus (HSV) families are broadly studied [1], and Bernard Roizman continues to be the undisputed “father” of herpes virology. Regarding HSV latency and the subject of this perspective, Jack Stevens and colleagues were primary players in the field of neurovirulence and latent infection using classical virological approaches [2,3,4,5]. However, in the early 1980s and during the dawn of molecular biology, Nigel Fraser and associates were the first to use modern molecular biology techniques to understand HSV-1 latency [6,7,8].

**Figure 1:**
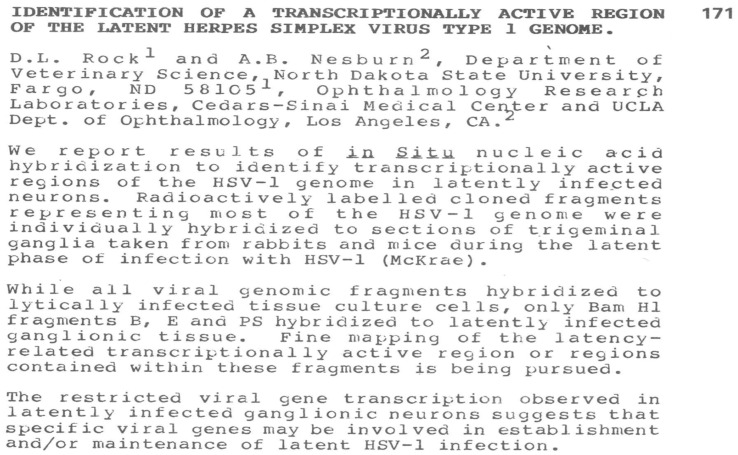
The abstract and poster presented by D.L. Rock and A.B. Nesburn at the IHW meeting regarding LR-RNA.

Part 1: First reported detection of latency-related RNA (LR-RNA) in trigeminal ganglia (TG) of latently infected rabbits. After joining North Dakota State University as an Assistant Professor and in collaboration with Anthony Nesburn, Dan Rock presented an abstract at the 11th International Herpesvirus Workshop (IHW) meeting in Leeds, England (21–26 July 1986). A copy of this abstract is shown below, describing the first reported detection of LR-RNA in TG of latently infected rabbits, and this transcript was antisense to ICP0 (Figure 1).

**Figure 2:**
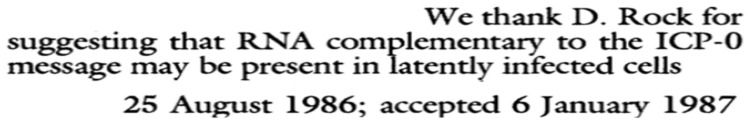
The abstract and poster presented to acknowledge D. L. Rock at the IHW meeting.

The above abstract and poster presented at the IHW meeting clearly demonstrated that LR-RNA (now named LAT) is antisense to ICP0, and this study was later published in the *Journal of Virology* [9]. However, prior to publishing this study, Stevens et al. [10] published a paper in *Science* reporting that an RNA complementary to a herpesvirus alpha gene mRNA is prominently expressed in latently infected neurons. In their acknowledgment, they thanked Dan Rock for “*suggesting that RNA complementary to the ICP0 message may be present in latently infected cells*”, as shown in this statement copied from the Stevens et al. paper [10] (Figure 2).

The abstract and poster that Dan Rock presented at the 1986 IHW, as well as his published study [9], clearly indicated that LR-RNA is antisense to ICP0 using single-stranded DNA probes prepared from recombinant M13 clones containing HSV-1 fragments (see D. Rock abstract, above). The most intriguing part of Stevens’ *Science* paper was that the 11th IHW ended on 26 July and the Stevens paper was submitted on 25 August 1986 (31 days after the end of the IHW meeting) (see submission date of Stevens’ 1987 *Science* paper above). Stevens et al. [10] indicated that they cloned a fragment of HSV-1 into M13 phage vectors, which they named mp8 and mp9, each expressing one strand of the ICP0 region. Thus, in 31 days, Stevens et al. [10] constructed the insert and cloned it into two phage plasmids, prepared single-stranded DNA, performed and replicated experiments, wrote the manuscript, and submitted it to *Science*.

Part 2: Role of LAT in apoptosis. We previously reported that significantly higher levels of apoptosis were detected in rabbits acutely infected with an LAT null mutant virus (dLAT2903) than in rabbits similarly infected with the parental WT HSV-1 McKrae strain [11]. This study also demonstrated that LAT inhibited apoptosis in transient transfection studies, which indicates that LAT directly impairs apoptosis. Our paper reporting this result was published in *Science* in 2000. One group wrote to *Science* to complain that our work was not correct. *Science* published this letter, which can be found in a search of PubMed [12], while our response letter is not available on PubMed but did appear at the end of their letter. In subsequent years, multiple groups have confirmed that LAT has anti-apoptotic functions [13,14,15,16,17,18]. Arguments that LAT is unable to inhibit apoptosis were further proven by generating recombinant viruses expressing cellular anti-apoptosis genes in place of LAT, and these viruses restored wt reactivation [19,20].

Because these cellular genes have only one function, they inhibit apoptosis, this provides compelling evidence that LAT does inhibit apoptosis and this function is important for reactivation. Ultimately, the same group that claimed LAT is not involved in blocking apoptosis [12] reported that LAT promotes neuronal survival [14]. Thus, it appears that for some, “half-empty” is different from “half-full”!

Another neurotropic herpesvirus, bovine herpesvirus 1 (BoHV-1), encodes an LAT-like transcript (LR gene) that is abundantly expressed during latency, which encodes a protein (ORF2) that inhibits apoptosis in transient transfection assays [21,22]. Additional studies revealed a mutation, where ORF2 was mutated, that did not reactivate from latency in calves, and this mutant induced higher levels of TG neurons that underwent apoptosis [23,24]. Finally, when the wt LR gene was inserted into an LAT null mutant (dLAT2903), the wild-type levels of reactivation from latency were restored [25,26]. Currently, HSV-1 strains RE, KOS and McKrae are used in the mouse models of ocular HSV-1 infection. Although they are different from each other in terms of their virulence, there is no difference in LAT expression and functions based on the virulence of HSV-1 strains.

Part 3: The role of LAT in CD8^+^ T cell exhaustion. In a groundbreaking 2006 study, Rafi Ahmed and colleagues demonstrated the role of PD-1/PD-L1/PD-L2 in CD8^+^ T cell exhaustion [27]. The exhausted CD8^+^ T cells had significantly reduced function, which is consistent with CD8^+^ T cell proliferation and exhaustion in response to continued long-term exposure to T cell-specific antigens [28,29,30]. Based on these studies, we too asked if, like LCMV, continuous LAT expression during latency influenced T cell exhaustion in the TG of latently infected mice. We included our preliminary data in our grant renewal and the primary reviewer of our grant wrote two pages describing how our data are counter to their published studies suggesting a role for CD8^+^ T cells in maintaining latency [31,32]. However, we and others later used LAT(+) and LAT(−) viruses to show that the presence of LAT leads to the generation of dysfunctional CD8^+^ T cells in the TG of latently infected mice [33,34]. Our published study using LAT(+) and LAT(−) viruses as well as PD-1^−/−^, PD-L1^−/−^, and PD-L2^−/−^ mice clearly demonstrated that LAT expression contributes to CD8^+^, but not CD4^+^ T cell exhaustion [33]. Later, Benmohamed’s group used LAT(+) and LAT(−) viruses and extensive FACS analysis to confirm our results, showing that LAT contributes to CD8^+^ T cell exhaustion [34]. Two years later, another group confirmed both our studies and those of Benmohamed [35].

Part 4: Involvement of CD8α^+^ DCs and not CD8^+^ T cells in HSV-1 latency. In the above studies, we and others clearly demonstrated that, consistent with the pioneering study using LCMV, CD8^+^ T cells are not completely functional in vivo [27,33,34]. However, between 2000 and 2003, several papers were published showing that CD8^+^ T cells infiltrate into TG at the time of HSV-1 establishment of latency and inhibit reactivation from latency [31]. Furthermore, a subset of CD8^+^ T cells remain in direct contact with infected neurons [32]. Using ex vivo cultures of latent TG, the authors showed that CD8^+^ T cells can block HSV-1 reactivation from latency [31,32]. These reports showed the importance of CD8^+^ T cells in maintaining latency using ex vivo studies while published studies of ours and others suggested that CD8^+^ T cells are not functional in vivo. Thus, to evaluate the contribution of CD8^+^ T cells in maintaining latency, we used CD8α^−/−^, CD8β^−/−^, and β2M^−/−^ mice and demonstrated that CD8^+^α DCs, but not CD8^+^ T cells, promote the maintenance of latency [36,37]. Overall, the detection of infiltrates in the TG of latently infected mice most likely indicates subclinical or unsuccessful reactivation by the virus when low levels of lytic cycle proteins are expressed. Regardless of which scenario is correct, CD8^+^α DCs, and not CD8^+^ T cells, play an essential role in regulating the maintenance of latency. The discrepancy between our results and those of other studies, regarding the importance of CD8^+^α DCs versus CD8^+^ T cells in maintaining latency, is probably due to the use of anti-CD8 antibodies in ex vivo studies. Anti-CD8 antibody detects CD8α cells, thus depleting both CD8^+^α DCs and CD8^+^ T cells. However, our studies used a combination of knockout mice and transfer experiments that clearly demonstrated the importance of CD8^+^α DCs and not CD8^+^ T cells.

It was previously reported that blocking reactivation from latency in ex vivo TG cultures by CD8^+^ T cells is mediated at least in part by gamma interferon (IFNγ) [38]. The authors showed that neutralization of IFNγ significantly enhanced the rate of HSV-1 reactivation from latency in ex vivo TG cultures. In contrast to this study, we previously did not detect any differences in latency levels using IFNγ or a recombinant HSV-1 expressing IFNγ (HSV-IFNγ) under control of the LAT promoter [39,40,41]. Replication of HSV-IFNγ was wild type in tissue culture and mouse eyes and its expression did not affect latency levels despite IFNγ expression during latency. Thus, our published studies using knockout mice and a recombinant virus continuously expressing IFNγ in TG of latently infected mice rule out the involvement of IFNγ in latency.

We were the first group to show the presence of LAT in TG isolated from cadavers [41]. Based on the role of CD8^+^ T cells in maintaining latency in mice [32], the presence of CD8^+^ T cells in the TG of cadavers was later reported using human TG [42]. However, these CD8^+^ T cells may be (1) migratory due to the absence of perfusion; (2) associated with reactivation, as shown in rabbit, or subclinical reactivation, as shown in mice; (3) associated with the status of the individual before death; (4) associated with trauma, thus death that is more severe than reactivation associated with stress or sun exposure; or (5) associated with the presence of other infectious agents (i.e., viral, bacterial, etc.); also, the prolonged time before the TG were obtained from cadavers could have complicated these studies. Thus, except for the relevant presence of HSV LAT or DNA in human TG, detection of infiltrates may not indicate what really happens during latency. In most published studies, different groups examined the role of CD8^+^ T cells in maintaining latency by FACS or IHC. Ironically, these studies used an anti-CD8α antibody that detects both CD8α^+^ DCs and CD8α^+^ T cells.

Part 5: Detecting LAT in immune cells. Previous studies using IHC, EM, and in situ nucleic acid hybridization showed that neurons of latently infected mice, rabbits, and humans express LAT [9,42,43,44,45,46,47,48,49,50,51,52,53,54,55,56,57,58,59,60,61,62,63,64]. After the first report detecting LR-RNA in TG of latently infected rabbits in 1986, LAT/LR-RNA was generally believed to be the only HSV-1 transcript detected in the TG of latently infected hosts. However, the development of more sensitive molecular techniques enabled the detection of small amounts of other viral gene products in neurons of latently infected mice [47,48,49]. We also showed that TG from LAT(+)-infected mice had significantly higher viral antigen levels than LAT(-) TG during latency [33]. These findings suggest either a low-level expression of viral antigens in a small number of neurons during latency, or that subclinical or abortive reactivations occur in mice at very low levels. These “subclinical” reactivation episodes could be due to virus reactivation in neurons of latently infected mice and/or other cell types in the TG of latently infected mice. We tested this hypothesis by isolating CD45^+^ cells from the TG of latently infected mice and using RT-PCR to compare LAT expression in these cells with neurons of latently infected mice [50]. In this study, we detected LAT in immune cells, but at lower levels than in neurons of latently infected mice. Based on RT-PCR results and single-cell analysis, we examined various immune cells in the TG of latently infected mice, showing the presence of LAT in B cells, DCs, fibroblasts, glial cells, innate lymphoid cells (ILCs), macrophages, microglia, monocytes, NK cells, neurons, neutrophils, CD4^−^CD8^−^, CD4^+^, and CD8^+^ T cells [50]. Due to the uniqueness and novelty of our findings and our previous experience with unfounded accusations that LAT does not play a role in apoptosis or CD8^+^ T cell exhaustion, we performed a second set of single-cell transcriptomic experiments on the TG of latently infected mice and obtained the same results. Previous studies using single-cell analysis typically perform one experiment rather than multiple experiments because the number of cells used for analysis is a crucial factor [51,52,53,54,55]. Thus, even though the normal standard is single-cell RNA-sequencing (sncRNAs) analysis, we performed a second sncRNAs analysis. Our *Science Advances* paper provides solid evidence that gB and gD DNA are present in CD4^+^, CD8^+^, DC, and ILC cells [50]. Consistent with our findings, a 2022 paper published in the Cell Press Journal, *Neuron*, from a Harvard group, detected HSV-1 LAT in immune cells and fibroblasts of human TG [56]. Our published study in mice [50] and Yang’s study in humans [56] used LAT as a marker of latent TG and we identified infected cells based on LAT expression. However, many isolated cells may be infected but do not express LAT, especially non-neuronal cells, as previous reports showed that these cells establish latency in the presence of acyclovir but do not express LAT [57].

Not to our surprise and with the nature of working with LAT, even before the ink had dried on our paper, a group contacted *Science Advances* questioning the validity of our results without performing any experiments, but rather based on questionable reanalyses of our RNA-seq data. This group’s concern with our detection of LAT in immune cells, as I was told by one of the authors of the *Science Advances* inquiry, was that our work would negatively impact their research. The authors of this inquiry failed to consider our data showing LAT RNA detection in isolated immune cells or the presence of gB and gD DNA in isolated CD4^+^, CD8^+^, DC^+^, and ILCs cells. They also ignored a publication showing the detection of HSV-1 LAT in human TG [56]. Our *Science Advances* paper came online 23 January 2023. Reasonable approaches to prove or disprove our work and, by extension, the work of Yang et al. [56], would have been to repeat our experiments and independently test whether our studies are reproducible. The group that contested our results had over a year to repeat our experiments many times over; however, their goal was not to prove our work by repeating our work. Although scientists do not always agree, this group could have performed the experiments and presented their data to support or negate our results rather than misrepresenting our work in the absence of alternative evidence. These “scientists” spent more than a year Journal shopping to publish their opinion without experimental evidence. As history and current times have shown, people who lack new ideas, dislike “new ideas”, as we have seen in different forms and at different times.

Part 6: Detecting reactivation in isolated immune cells. The main characteristics of HSV-1 infection are (1) establishment of latency and (2) during the latent phase of infection, and in contrast to primary infection, LAT is the only gene product expressed abundantly in infected mice, rabbits, and humans [9,42,44,45,46]. Although additional HSV-1 transcripts have been detected, they are expressed at significantly lower levels than LAT transcripts [47,48,58]. The HSV-1 LAT gene is known to play a critical role in enhancing the reactivation phenotype [44,59,60,61] and LAT deletion mutants have a significantly lower reactivation phenotype in mice and rabbits [44,59,60,61]. Although spontaneous reactivation occurs in rabbits at levels similar to that seen in humans [62], spontaneous reactivation in a murine model of ocular HSV-1 is rare. Following ocular infection, latent HSV genomes express LAT in a portion of those neurons maintaining them, and viruses can be recovered by co-cultivating explanted ganglia [63,64,65,66,67,68,69,70,71,72,73]. An interesting variation of this method, which comes closer to an in vivo method, has been developed using viruses that can be reactivated in latently infected mice after transient exposure to UV-B radiation [74] or hyperthermia [75].

To determine if reactivation occurs in isolated immune cells from infected TG, mice were infected with HSV-1 strain McKrae, as we described previously [50]. On day 28 post-infection, infected TG were isolated, and TG from four mice were pooled, digested with collagenase D, and dissociated into single-cell suspensions. The cells were then separated using a Percoll density gradient. The top cell layer, containing neurons, was discarded while the middle layer, containing immune cells, was collected and co-cultured with Vero cells to detect CPE as a measure of reactivation. Of the four samples from 16 mice, all four were reactivated, demonstrating that HSV-1 can be reactivated from this cell layer in which neurons were removed by the density gradient. We then used two separate approaches to measure reactivation in isolated immune cells from the TG of infected mice (unpublished data). First, we determined which immune cell types can be reactivated, then stained cells from the middle layer of the density gradient with BV510 anti-CD45, BV421 anti-CD3, and 7-AAD. CD45^+^CD3^+^ T cells and CD45^+^CD3^−^ non-T immune cells were sorted using a BD Aria 3 cell sorter. Each cell type was then co-cultured with Vero cells to assess HSV-1 reactivation. In two independent experiments (a total of 19 mice, with 9 or 10 TG pooled for each group), no HSV-1 reactivation was seen in either the T cell group, or non-T cell group. The lack of reactivation in these sorted cells could be due to cell damage during sorting or insufficient cell numbers (approximately 20,000 cells per group). However, we previously detected LAT RNA, as well as LAT, gB, and gD DNA, in isolated CD4, CD8, macrophage, and DC cells [50]. Further investigation is needed to determine whether HSV-1 can be reactivated in specific immune cell types using a cell isolation column rather than FACS. Second, we isolated immune cells as described above using 15 mice (six TG per sample). The collected middle layer cells were stained with anti-CD45 antibody, and positive cells were purified using beads per the STEMCELL Technologies protocol. Purified CD45^+^ cells were then co-cultured with Vero cells and monitored for 3–7 days for CPE. Four of five (80%) samples reactivated in two independent experiments, indicating that HSV-1 can reactivate from purified immune cells. These results suggest that reactivation can occur in isolated immune cells, depending on how cells are isolated. Studies are in progress to determine reactivation in individual cell populations using density gradient and staining with each cell type. In the above explant reactivation of infected immune cells, we made sure that the isolated immune cells did not contain neurons.

Part 7: In vitro versus in vivo models of latency and concerns about using neuronal cells to study the HSV-1 latency reactivation cycle. After discovering LAT, Wilcox and Johnson [76] were the first to use an in vitro model of latency in the presence of acyclovir. Between the late 1980s and early 1990s, other groups used C1300, Neuro2A, PC12 and many other cell types to study HSV-1 latency in vitro in the presence of antivirals. With improved neuronal isolation and culturing techniques in late 2000, some groups began to again use the in vitro model of latency. Infection of neurons by HSV-1 produces low levels of virus and cell death unless acyclovir is added to cultures. However, acyclovir treatment causes gaps in replicating viral DNA, which may increase transcription and “reactivation” in latently infected neuronal cultures. Further, LAT is not abundantly expressed in most “latently” infected neuronal cell culture models of latency. This is a problem because it is well documented that LAT is required to establish and maintain latency in mouse and rabbit infection models [9,42,44,45,46]. Only one published study has shown that LAT is important in neuronal models of latency using Lund human mesencephalic (LUHMES) cells in vitro [77]. Most neuronal models of latency do not use sensory neurons and thus cannot be correlated with in vivo latency models. In contrast to in vitro latency models, in which infected cells die without antiviral treatment, in vivo, neurons survive infection and establish and maintain latent infection in the host surrounded by non-neuronal cells that play critical roles in signaling to neurons in vivo.

Non-neuronal cells are not present in in vitro neuronal cultures used to study latency, but infiltrating immune cells are readily detected in human and mouse latency models [50,56]. Non-neuronal cells secrete cytokines that impair reactivation from latency and impair viral gene expression to promote latency establishment and maintenance. The absence of these immune cells in neuronal models of latency reduces the biological relevance of these models. In one latency model, used by the Cliffe–Wilson groups, after induced reactivation, viral gene expression occurred 12–18 h post reactivation [78], while in explant-induced reactivation, viral protein expression (ICP0, ICP4, and VP16) was detected within 8 h after explant using IHC [79]. Although it is unlikely that two phases of reactivation exist in vivo, it seems to occur in several in vitro models of latency [80,81]. Thus, detecting LAT in different immune cells from human TG is further proof that the in vitro model of latency in the presence of acyclovir or similar antiviral drug classes and in the absence of immune cells is not consistent with the in vivo model of latency. Thus, use of these “neuronal models” are limited and do not reflect the complexity of in vivo situations, even in mouse models of infection. Of note, latency-like conditions can be established in any non-neuronal cultured cell lines when cultures are incubated with acyclovir [57]. Although many articles have been published on different latency models, a detailed review of latency models was recently published by Dr. David Leib’s group [81]. The authors concluded that different in vitro latency models are not consistent with, or relevant to, in vivo latency models. While in vivo models of latency may not be perfect, they are more biologically relevant than in vitro cultures of HSV-1 latency.

## 2. Conclusions

The above perspective summarizes 40 years of work related to LR-RNA/LAT with many of the ups and downs, controversies, backstabbing, and manipulation by the old and new guard. Thus, we should expect more of the same in the next 40 years or until the complexity of LAT functions are solved. After attending an NIH study section, a prominent viral immunologist commented that ‘herpes-virologists eat each other’ if you are not part of their club. We might also add that when it comes to herpes latency, some in the crowd smother competition with significant efforts to stop research funding. One example that I heard directly, along with another investigator, occurred during the 9th Annual Symposium of the Colorado α-herpesvirus Latency Symposium (CALS) held on 8–11 May 2019, in Vail, CO, where during a conference dinner, a permanent member of one of the two main virology study sections promised two individuals that they would help them with their grants. Both of these investigators’ grants were reviewed in the permanent member’s study section and were funded. I and another virologist looked at each other and could not believe what was publicly stated. The same individual aggressively approached an ad hoc reviewer in the presence of other reviewers asking why the ad hoc reviewer was so tough on the grant of one of the individual’s friends and if there was any room for negotiation. Even out of the spotlight, scientists do not discuss this type of issue so openly. We all thought this individual felt they were above the rules and did not need to comply with the ethics of science! Thus, the behavior of some herpes virologists recalls what Rosalind Franklin endured when she was denied recognition for her role in unraveling the structure of DNA. As with athletics, competition in science needs to be on a level playing field. It is often difficult to determine who was the first to discover critical issues in science and in the end, for humankind if not for history, it is not important. However, it is unfair and offensive when a small group succeeds in suppressing the input of outsiders, particularly in an environment where staying active requires robust support. Thus, when it comes to novel findings about LAT, a diligent literature review will establish, even to the most novice investigator, the timeline of discovery and reproducibly (or not) of findings to the relevant scientific community. Fortunately, in the past 30-plus years, my NIH fundings have escaped the toxicity of many of these individuals since, after all, the world has more honest, fair, and balanced people.
